# Global WEIRDing: transitions in wild plant knowledge and treatment preferences in Congo hunter–gatherers

**DOI:** 10.1017/ehs.2020.26

**Published:** 2020-06-01

**Authors:** Gul Deniz Salali, Mark Dyble, Nikhil Chaudhary, Gaurav Sikka, Inez Derkx, Sarai M. Keestra, Daniel Smith, James Thompson, Lucio Vinicius, Andrea Bamberg Migliano

**Affiliations:** 1 Department of Anthropology, University College London, London WC1H 0BW, UK; 2 Leverhulme Centre for Human Evolutionary Studies, Department of Archaeology, University of Cambridge, Cambridge, CB2 1QH, UK; 3 Department of Anthropology, University of Zurich, 8057 Zürich, Switzerland; 4 Department of Anthropology, Durham University, Durham DH1 3LE, UK; 5 Bristol Medical School: Population Health Sciences, University of Bristol, Bristol BS8 2BN, UK

**Keywords:** Cultural evolution, African BaYaka Pygmies, Traditional knowledge, Indigenous health, Cultural change

## Abstract

Cultures around the world are converging as populations become more connected. On the one hand this increased connectedness can promote the recombination of existing cultural practices to generate new ones, but on the other it may lead to the replacement of traditional practices and *global WEIRDing*. Here we examine the process and causes of changes in cultural traits concerning wild plant knowledge in Mbendjele BaYaka hunter–gatherers from Congo. Our results show that the BaYaka who were born in town reported knowing and using fewer plants than the BaYaka who were born in forest camps. Plant uses lost in the town-born BaYaka related to medicine. Unlike the forest-born participants, the town-born BaYaka preferred Western medicine over traditional practices, suggesting that the observed decline of plant knowledge and use is the result of replacement of cultural practices with the new products of cumulative culture.

## Graphical abstract

**Figure:**
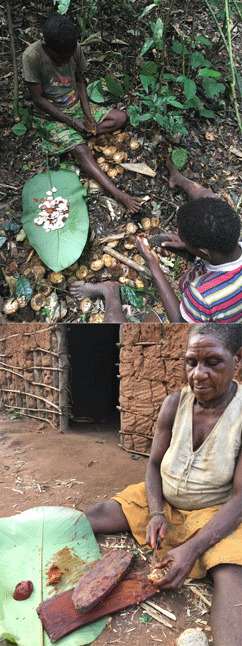

**Media summary:** Is the world getting WEIRDer? Town-born hunter–gatherers in Congo use fewer wild plants and prefer Western over traditional medicine.

## Introduction

Hunting and gathering was the primary mode of subsistence for most of human history. Studies of extant hunter–gatherers, therefore, may help us understand the context in which characteristic human traits have evolved. Nevertheless, human behaviour research has been dominated by studies in WEIRD (Western, Educated, Industrialized, Rich and Democratic) societies (Henrich, Heine, & Norenzayan, 2010). While there is a growing agreement on the necessity of studies on non-WEIRD populations, cultural practices in many areas, even in the remotest regions, are converging owing to globalization (Rozin, 2010). This process of cultural convergence has been appropriately coined as Global WEIRDing (Cooperrider, 2019). Research on current day hunter–gatherers is exciting not only because these populations represent a hunting–gathering way of life, but also because they can provide insights into how humans adapt to rapid changes in their environment and the processes of global WEIRDing.

Mbendjele BaYaka hunter–gatherers are one of the few remaining hunter–gatherer groups and they exhibit variation in their level of market integration. Some BaYaka live semi-permanently or permanently in large settlements near farmer villages or market towns. The BaYaka living in towns engage in wage-labour more frequently and have greater financial capital (as indicated in the amount of bride-price paid) than those living further away from logging towns (Salali & Migliano, 2015). They are also more future-oriented (Salali & Migliano, 2015), and spend less time hunting using traditional techniques (such as with spears) or honey collecting (Riddell, 2013). Moreover, the actual forest space around the logging villages is reduced owing to rapid deforestation in these areas (Laporte, Stabach, Grosch, Lin, & Goetz, 2007). The variation in the mode of living of different BaYaka groups provides a unique natural experiment where behavioural adaptations to the hunting and gathering niche, and to the rapidly changing environments, can be studied.

The BaYaka rely on plants for food, medicines, foraging and cultural practices (Salali et al., 2016). They are famous for their knowledge of medicinal plants and healing abilities among their neighbouring groups (Bahuchet, 2014). Knowledge of wild plants is important because in addition to substantially contributing to diet (Crittenden & Schnorr, 2017), plants may provide health benefits to knowledge holders in the absence of Western medicine. For example, mothers with a greater body of knowledge about plants are found to have healthier children in Tsimane’ forager–horticulturalists, independent of potentially confounding variables related to education, market integration and acculturation (McDade et al., 2007). In a previous study, we have shown that BaYaka mothers who use more medicinal plants to treat respiratory system disorders have children with higher body mass index (Salali et al., 2016). The BaYaka also give a special value to wild plant knowledge. While material goods such as food, tools and clothes are shared freely, certain knowledge of the medicinal uses of plants is exchanged for goods and money (Lewis, 2015). Given the importance of wild plants in forest hunter–gatherers, it is crucial to understand what happens to the accumulated plant repertoire when populations undergo rapid transitions owing to globalization.

Studies on the effects of globalization on local ecological knowledge have documented the loss of cultural traits within a generation (Godoy & Reyes-García, 2009; Gomez-Baggethun, Mingorria, Reyes-García, Calvet, & Montes, 2010; Reyes-García, Guèze, et al., 2013; Reyes-García, Luz, et al., 2013). In this paper, we aim to examine if a similar decline in ecological knowledge occurs in the BaYaka, and if so, what factors drive the cultural change. Specifically, we ask whether changes in cultural traits happen owing to the changes in individual preferences towards new cultural practices via the process of global WEIRDing. We do this by comparing the knowledge and use of wild plants, and preferences in treatment methods among town-born and forest-born BaYaka groups.

## Methods

### Study population

Mbendjele hunter–gatherers are a subgroup of the BaYaka (in literature sometimes referred as African Pygmies) who speak the Mbendjele language (Lewis, 2014). They reside across the forests of the Republic of Congo. BaYaka subsist by hunting, trapping, fishing and gathering forest products such as wild yams, caterpillars and honey. They also engage in trade with neighbouring groups and wage labour (for farmers and logging companies). The frequency of trade and wage labour varies depending on the availability of trading opportunities such as the presence of Bantu traders or proximity to market towns (Salali & Migliano, 2015). The BaYaka traditionally live in multi-family camps of 10–60 individuals (Dyble et al., 2015). Nevertheless, semi-sedentary BaYaka camps in market towns can reach over 200 individuals. The BaYaka are predominantly exogamous (either father or mother coming from a different clan) and serially monogamous, although there are a few cases of polygyny (Chaudhary et al., 2015). BaYaka move between different campsites depending on the availability of food resources and trading opportunities with villagers. Visits by friends and families from other camps are also common.

### Measuring plant knowledge and use

Between April and August 2014, the first author and her BaYaka translator (who translated from French to Mbendjele) asked 15 adult informants (10 men, five women) to list the names of plants they used for medicinal purposes. She then chose a subset of 33 plants that are used by the population and asked another 219 individuals (101 men, 118 women) across four campsites whether they knew each of the 33 species, and if so, whether they used it for any purpose. (‘Do you know plant “x”?’ If the participant replies yes, ‘Do you use it?’ If the participant says yes, ‘What do you use it for?’. See Supplementary Information for further details.) We calculated the total number of plants that were reported to be known (plant knowledge score) and used (plant use score). For example, if a participant reported knowing 15 out of 33 plants, her knowledge score was 15. If among those 15 plants, she reported using 10, then her plant use score was 10. The use of a cumulative score such as this one is most appropriate where plant knowledge falls onto a single continuum of expertise where individuals with a lower plant knowledge score know a subset of plants known by more knowledgeable individuals, rather than a smaller but more specialist set of plants unknown to those with higher overall scores, for example. In order to check this assumption, we plotted the probability of knowledge of each individual plant against a single latent axis of ‘knowledge’ derived from a simple item response theory model without fixed effects (Bunce & McElreath, 2017). These plots show clear positive relationships between knowledge of each plant and a single latent axis (Figure S1), suggesting that plant knowledge does indeed fall along a single continuum.

The 2014 fieldwork took place in the Likouala and Sangha regions of Congo's Ndoki forest, where G.D.S, N.C. and J.T. stayed in four camps, three of which were located in the forest and one of which was located in a logging town. The researchers stayed in each camp between 10 days and a month and at the end of their stay gave each household a gift (either a pot, a machete or a head torch) for their hospitality and participation in research. The forest camps were located by the mud roads cutting through the dense forest opened by a logging company in the late 1990s. As well as hunting and gathering, individuals in these camps trade forest products with farmers for cultivated food, alcohol and cigarettes. They frequently move to deeper parts of the forest depending on the availability of food resources (e.g. for fishing in the dry season between December and March) or the presence of a land-related conflict with non-BaYaka groups.

The town camp was located in Pokola, a logging town in Northern Congo where economic opportunities attract workers and traders from both the local area and internationally, from countries such as Cameroon and Chad. The presence of a hospital offering free health care run by the logging company located in Pokola also attracts people into the town. The BaYaka camp in Pokola consists of individuals who are born and raised in the town, and families who temporarily move to the town and reside there to visit families or for ceremonies, engage in wage labour, buy products such as clothes or machetes in the market or use the hospital. The BaYaka camp in town is a few kilometres away from the town centre, closer to the surrounding forests. Although the BaYaka living in the town engage in wage labour more frequently than the BaYaka coming from forest regions (Salali & Migliano, 2015), they still go on daily foraging trips and stay in the forest temporarily in fishing and caterpillar seasons.

We had information on the birth places of 88% of the participants (*n =* 193). The majority of the participants in forest camps were from the same birth region: 91% of the participants from the forest camps 1 and 2 were born in Minganga region and referred to themselves as ‘Mbendjele of Minganga’; 97% of the participants from forest camp 3 were born in Ibamba region and known as the ‘Mbendjele of Ibamba’. The participants residing in the town camp were born in different regions (Supplementary Figure S2). Some of the participants in the town were born and raised there, whereas some families had migrated to town either temporally or permanently from other regions, including Minganga and Ibamba (Supplementary Figure S2).

### Treatment method and preference

We returned to the same region in July–August 2018 to further examine treatment methods and preferences in BaYaka born and raised in the logging town or in forest regions. We returned to the town camp in Pokola and forest camp 1 (Longa) and visited a new camp located an hour's drive from forest camp 1 (see Table 1 for the camp names, data collection period and participant age and gender). Because the camp composition is fluid with individuals moving between different campsites, there were familiar faces as well as new ones in the camps. For this reason, we refer to the two forest camps we visited in 2018 as forest camp a and forest camp b (Table 1). In the town, we targeted the BaYaka who were born and raised there and excluded individuals who reported temporarily moving from other regions to town. All of the town-born BaYaka interviewed were below 45 years of age, whereas participants in forest camps included individuals above 45 years.

**Table 1.** Camp location and names, data collection period and sample size

Camp location (name used in the paper)	Data collection period	Number of participants (number female)	Collected data
Longa (forest camp 1, Minganga region)	2014	59 (28)	Plant knowledge and use
Masia (forest camp 2, Minganga region)	2014	22 (12)	Plant knowledge and use
Ibamba (forest camp 3)	2014	31 (18)	Plant knowledge and use
Pokola (town camp)	2014	107 (60)	Plant knowledge and use
Longa (forest camp a, Minganga region)	2018	54 (28)	Treatment method and preference
Njoki (forest camp b, Minganga region)	2018	28 (17)	Treatment method and preference
Pokola (town camp)	2018	46 (26)	Treatment method and preference

We asked participants (aged >16 years) what they did for treatment when they are sick (treatment method). We followed this up by asking whether they preferred using traditional medicine or going to hospital (treatment preference). We used two separate questions because participants did not have equal access to Western medicine. The hospital in town is within walking distance for participants in the town, whereas those who reside in forest camps need to get and pay for transportation to reach the hospital. As such, we aimed to capture whether participants’ treatment method and preference differed owing to the limitations on their access to Western medicine. For the treatment method question, the participants’ replies included the following: ‘I go to hospital’, ‘I use traditional medicine’, ‘I do not do anything’, ‘I use pills’. For the treatment preference question, the replies were as follows: ‘I prefer going to hospital’, ‘I prefer traditional medicine’, ‘I prefer going to hospital first and if it does not work I use traditional medicine’, ‘I prefer using traditional medicine first and if it does not work I go to hospital’.

### Statistical analyses

To examine the effects of birth place on plant knowledge and use score we conducted ANOVAs controlling for the participants’ sex and age group. The response variable was either the plant knowledge or the use score, and the factorial predictors were: birth place (Minganga, *n =* 82; Ibamba, *n =* 54; Other, *n =* 28; Pokola (town), *n =* 29), sex (female, *n =* 104; male, *n =* 89) and age group (child, 5–15 years, *n =* 19; young adult, 15–25 years, *n =* 44; adult, 25–45 years, *n =* 72; old adult, 45+ years, *n =* 58). We used Tukey's honestly significant difference (HSD) tests to compare the means of knowledge and use scores among different levels of each factorial variable. Because the response variables were not normally distributed, we used the *transformTukey* function in R to transform the values based on Tukey's ladder of powers transformation (Mangiafico, 2019). We presented the results of the most parsimonious models obtained by model selection using the *anova* function in R.

We further investigated (a) if the BaYaka who were born in forest and later moved to town differed in their plant knowledge and use scores compared with the BaYaka who were born and residing in the forest and (b) if the BaYaka who were born in forest and later moved to town differed in their plant knowledge and use scores compared with the BaYaka who were born and residing in town. For the former analysis we excluded children and for the latter we excluded children and old adults owing to the small sample sizes (see Table 2 for sample sizes by age group, birth region and current camp residence). We conducted ANOVAs controlling for participants’ sex and age group, and used transformed plant knowledge and use scores for normality.

**Table 2.** Median plant use and knowledge scores (out of 33 plants) and sample size by birth place (forest vs. town) and current camp residence (forest vs. town)

	Median plant use scores			Median plant knowledge scores			Sample size		
	Forest born forest living	Forest born town living	Town born town living	Forest born forest living	Forest born town living	Town born town living	Forest born forest living	Forest born town living	Town born town living
**Child**	7	NA	9.5	18	NA	13	17	0	2
**Young adult**	18	11	13.5	29	21	17	23	5	16
**Adult**	25	19	17	30	31	23	41	21	10
**Old**	27	28.5	29	32	32	32	31	26	1
**Total**	23	22.5	15	30	31	20	112	52	29

We also examined how birth region, current camp residence, age and sex affected how a plant is used by using multiple correspondence analysis (MCA). MCA is a nonlinear multivariate analysis method that is mainly used to visualize data with categorical variables (Hoffman & Leeuw, 1992). Its principles are the same as in principal component analysis, except that the data consists of factorial variables. We performed MCA to visualize the clustering of individuals based on their uses of 33 plants using the R package FactoMineR (Le, Josse, & Husson, 2008).

To test whether reported treatment methods (hospital, traditional medicine, nothing, pills) and preference (hospital vs. traditional medicine) differed among the three BaYaka campsites we used pairwise proportion tests. We also conducted additional analyses with the subset of the forest sample with known ages to examine if age affected treatment method and preference. The data analyses were conducted using R statistical software. All data files and associated R codes are available at https://osf.io/udwzr/.

## Results

### Town-born BaYaka reported knowing and using fewer plants

**Figure 1.**
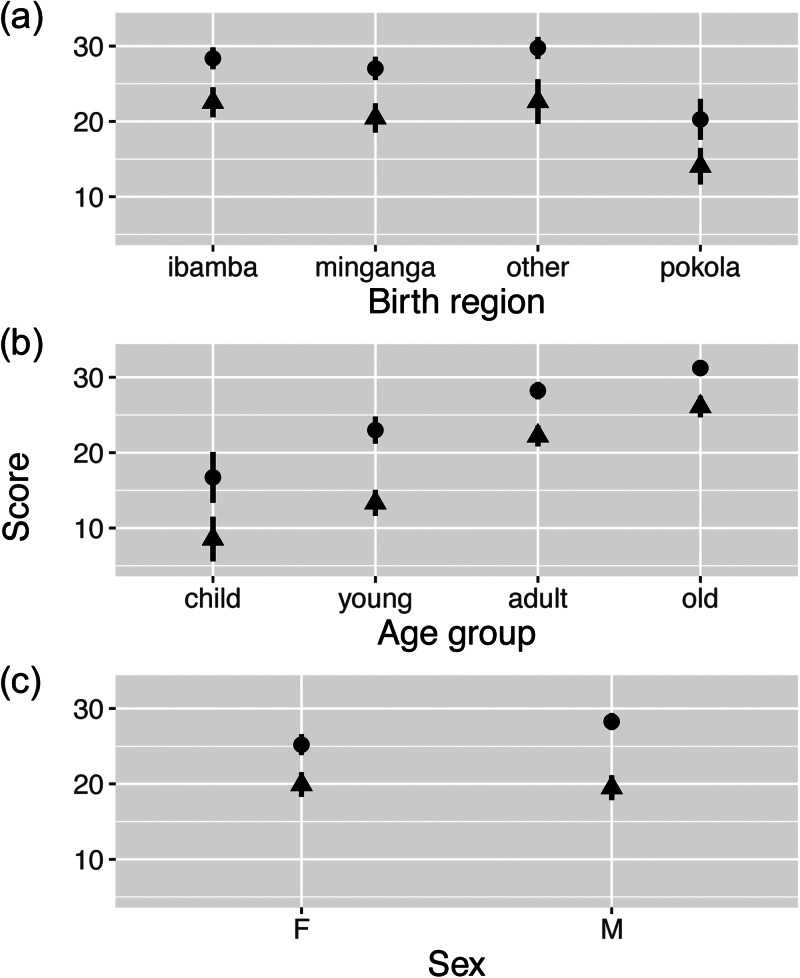
Mean plant knowledge and use scores by (a) birth region (Pokola is the logging town, and the other places are regions that include camps in the forest), (b) age group (child, 5–15 years; young adult, 15–25 years; adult, 25–45 years; old adult, 45+ years), (c) sex (F, female; M, male). Dots indicate plant knowledge score and triangles plant use score. Error bars show 95% confidence intervals.

Controlling for age and sex, people who were born in the logging town reported knowing fewer plants compared with people who were born in all other regions (Figure 1a; Table 3 Model 1–2; Tukey's HSD, *P* < 0.001). The median number of plants reported to be known was 20 (out of 33) for the town-born BaYaka, while the medians for participants from Minganga, Ibamba and other regions were 30, 30 and 31 respectively.

**Table 3.** ANOVA results of the effects of birth region, sex and age group on the number of plants that are known (Models 1–1, 1–2) and used (Models 2–1 to 2–3) by participants. Our analysis of model selection indicated that the models 1–2 and 2–3 were the most parsimonious ones

	*Knowledge score*							
	Model 1–1				Model 1–2			
	D.f.	*F*-Value	*P*-Value	Partial eta squared	D.f.	*F*-Value	*P*-Value	Partial eta squared
**Birth region (Ibamba, Minganga, Other, Pokola)**	3	19.57	<0.001	0.11	3	19.94	<0.001	0.10
**Sex (female, male)**	1	12.54	<0.001	0.09	1	12.78	<0.001	0.08
**Age group (child, young, adult, old adult)**	3	39.75	<0.001	0.40	3	40.51	<0.001	0.40
**Birth region × age group**	8	0.57	0.80	0.02				
***N***	193				193			

	*Use score*										
	Model 2–1				Model 2–2				Model 2–3		
	D.f.	*F*-Value	*P*-Value	Partial eta squared	D.f.	*F*-Value	*P*-Value	Partial eta squared	D.f.	*F*-Value	*P*-Value
**Birth region (Ibamba, Minganga, Other, Pokola)**	3	15.46	<0.001	0.08	3	15.2	<0.001	0.07	3	15.23	<0.001
**Sex (female, male)**	1	0.47	0.50	0.00	1	0.5	0.50	0.00			
**Age group (child, young, adult, old adult)**	3	49.31	<0.001	0.46	3	48.3	<0.001	0.44	3	48.71	<0.001
**Birth region × age group**	8	1.47	0.17	0.06							
***N***	193				193				193		

The BaYaka who were born in the town not only knew but also used fewer plants (Figure 1a; Table 3, Model 2–3; Tukey's HSD, *P* < 0.001). While the median number of plants reported to be used was 15 for the town-born BaYaka, the medians for people from Minganga, Ibamba and other regions were 21.5, 23.5 and 25 plants, respectively. Participants who were born in the two forest regions and the other unvisited regions did not differ in their plant use score (Tukey's HSD, *P =* 0.23 for Minganga-Ibamba, *P =* 0.99 for Ibamba-other, *P =* 0.37 for Minganga-other).

Our control variables showed that the BaYaka knew and used more plants as they got older (Figure 1b, Table 3). We also examined birth region × age interaction to test whether the BaYaka in each age group differed in their plant knowledge and use depending on where they were born. Overall, birth region × age interaction was not a significant factor in explaining the variation in knowledge and use scores (Table 3). Although male participants reported knowing a slightly but significantly higher number of plants than female participants (Figure 1c, Table 3, Model 1–2), the number of plants they reported using did not differ (Figure 1c, Table 3, Model 2–2). We confirmed these findings with additional regression analyses (Supplementary Tables S3 and S4).

### Forest-born BaYaka who moved to town later have not lost their plant knowledge

Our further analysis comparing the BaYaka who were born in the forest and later moved to town with those who were born and currently residing in forest regions showed that they did not differ in plant knowledge and use scores (Table 2, Supplementary Table S1). Contrarily, the BaYaka who were born in town and residing there had lower knowledge and use scores compared with those who were born in forest, and moved to town later (Table 2, Supplementary Table S2).

### Town-born BaYaka reported using plants for medicine less frequently

We examined how plant use differed between BaYaka born in different regions. First, we conducted MCA on plant uses to explore the data on a two-dimensional plane. This analysis showed that individuals clustered on different coordinates depending on their birth region, indicating that the BaYaka who were born in the same region had more similar plant uses (Supplementary Figure S3).

**Figure 2.**
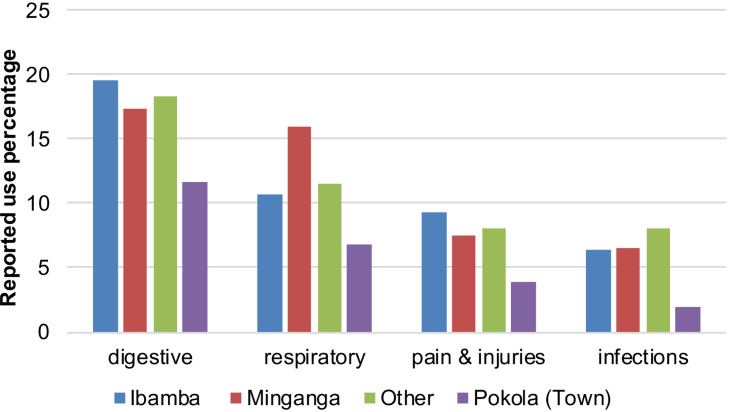
Reported plant use percentages for the treatment of most common diseases by birth place.

Participants who were born in town reported using plants for medicine less frequently (Figure 2). While almost 20% of the reported plant uses by participants from Ibamba region concerned treating digestive system disorders, only around 12% of the reported uses by town-born participants concerned treating those (Figure 2). Likewise, participants from forest regions reported using plants for treating respiratory disorders, infections and injuries more often than town-born participants (Figure 2).

We further investigated the plants that are frequently used by the forest-born BaYaka but reported as being used seldom by the town-born BaYaka. We first identified the plants that were used by less than half of the participants who were born in the town. Among those, we chose the ones that were frequently used (more than 50% of the participants reported using the plant) by the forest-dwelling BaYaka. In this way, we identified eight plants that were not reported as being used by the town-born participants but used by others. Table 4 shows the percentages of participants who reported using each of the eight plants as medicine with respect to their birthplace.

**Table 4.** The list of medicinal plants that are frequently used by the BaYaka who are born in forest regions, but not by the BaYaka who are born in the town

	Percentage of people using the plant as medicine by birth place			
Plant (BaYaka name)	Ibamba	Minganga	Other	Pokola (town)
**Bulaki**	72	63	63	14
**Indengo**	83	93	81	28
**Jongo**	50	54	33	10
**Kombo**	54	60	59	34
**Mopo**	44	49	67	21
**Moba**	76	67	70	41
**Mongo**	74	71	74	28
**Mokula**	52	61	56	17

### Town-born BaYaka reported using and preferring Western over traditional medicine

**Figure 3.**
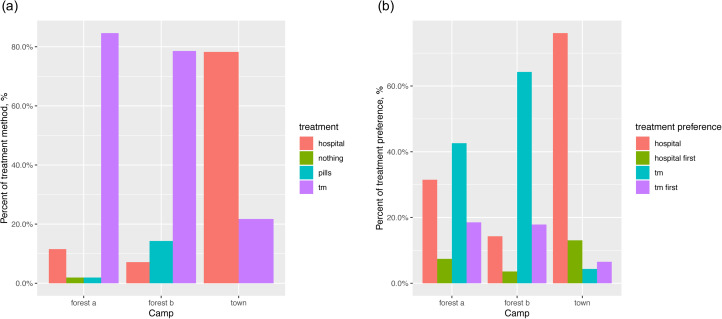
Reported percentages of (a) treatment method and (b) treatment preference by current camp residence. Hospital means participants reported going to hospital, or preferring hospital over traditional medicine; tm refers to traditional medicine. Pills are drugs bought from farmers or provided by health-aid projects. Hospital first corresponds to those responses where participants reported preferring hospital first, and if the treatment is not successful, using traditional medicine; tm first indicates preference for traditional medicine and hospital as a second choice if traditional practices do not work.

Almost 80% of the participants who were born in town reported going to hospital when sick, vs. only 11% of participants in forest camp a, and 7% in forest camp b (Figure 3a, 3-sample proportion test: *n =* 126, *χ*^2^_2_= 60.04, *P* < 0.001). More than 78% of the participants in forest camps reported using traditional medicine (i.e. wild plants) when sick, vs. only 22% of the town-born BaYaka (*n =* 126, *χ*^2^_2_ = 45.33, *P* < 0.001, Figure 3a).

When we asked participants whether they preferred going to hospital or using traditional medicine in times of sickness, 76% of those who were born in town reported preferring hospital, whereas only 31% of the participants in forest camp a and 14% in forest camp b preferred hospital over traditional medicine (Figure 3b, proportion test: *n =* 126, *χ*^2^_2_ = 32.73, *P* < 0.001). Some 18% of the participants in forest camps a and b and 6% of those in the town reported going to hospital as a second choice where traditional methods do not work (Figure 3b, proportion test: *n =* 126, *χ*^2^_2_ = 3.38, *P* = 0.18). On the other hand, 7% in the forest camp a, 3% in forest camp b and 13% in the town camp reported using traditional medicine as a second choice where Western medicine does not work (Figure 3b, proportion test: *n =* 126, *χ*^2^_2_ = 2.15, *P* = 0.34).

Our further analysis with the subset of the data indicated that age did not affect the use of and preference for traditional medicine in forest-born BaYaka (Supplementary Table S5–6).

## Discussion

Our results show that the BaYaka who were born in the town reported knowing and using fewer plants than the BaYaka who were born in forest regions. Plant uses that were lost in the town-born BaYaka concerned uses for medicine. Unlike the forest-born participants, the town-born BaYaka preferred Western medicine over traditional practices, suggesting that the observed decline of plant knowledge and use is the result of replacement of cultural practices with the new products of cumulative culture.

We showed that the BaYaka who were born in forest regions and moved to town had higher plant knowledge and use scores than those who were born in town. Our previous studies showed that medicinal plant knowledge is mainly shared within families in the BaYaka, but marital ties and strong friendships help connect distant families and promote knowledge exchange (Migliano et al., 2017; Salali et al., 2016). The observed difference in the plant uses of town-born and forest-born BaYaka living in the town may indicate little knowledge exchange among these individuals, perhaps owing to changes in social structure where connections among different households are weakened by the growing population size.

The observed decline in wild plant knowledge may also be due to reduced social learning opportunities. Almost all hunter–gatherer groups in the Congo Basin work as wage labourers in farmers’ fields in exchange for alcohol, agricultural food, cigarettes and other industrial products (Bahuchet & Guillaume, 1982; Oishi, 2012; Turnbull, 1961). Nevertheless, the frequency of such exchanges varies within and among those groups. In a previous study, we found that adults in the town camp engage in wage labour more frequently than the adults that reside in other camps (Salali & Migliano, 2015). Moreover, forests around the logging town Pokola are degraded by agricultural and logging activities that make the immediate environment less suitable for hunting and gathering (Laporte et al., 2007). These changes in subsistence activities and ecological conditions may result in BaYaka, growing up in towns and having fewer opportunities to observe and accompany adults during forest-related activities. This is especially important given that BaYaka children acquire forest skills through observation, imitation and practice (Lew-levy et al., 2019; Lew-Levy, Reckin, Lavi, Cristóbal-Azkarate, & Ellis-Davies, 2017; Salali et al., 2019). Nevertheless, during fieldwork we observed the BaYaka residing in the town going on long foraging trips, especially during fishing and caterpillar seasons, and when specific wild nuts are abundant. To conclude on whether town-born BaYaka have fewer social learning opportunities, data comparing the time spent in foraging in different BaYaka groups is needed.

Alternatively, the town-born BaYaka may be less motivated to learn about wild plants compared with the BaYaka who grew up in the forest. When we asked a young BaYaka man in town about living in the forest as opposed to living by logging roads or towns, we received the following answer: ‘It (solely living in the forest) was something of the past, something that our ancestors were doing’. The BaYaka are often humiliated and compared with animals by the neighbouring farmer populations because of their forest-oriented way of life (Lewis, 2005, 2014). We suspect that often, when the BaYaka make such claims about living in the forest, it is driven by the shame or negative feedback that they receive from farmers. This effect is much stronger for the young BaYaka living in towns as they interact with farmers on a daily basis. Nevertheless, they may not consider knowing about wild plants as being as valuable as learning French, which may allow them to better integrate in the society in ways that help them can get jobs and earn money.

Indeed, our findings suggested that town-born BaYaka who we interviewed in 2018 (all younger than 45 years) preferred going to hospital over using wild plants. In contrast, forest-born BaYaka, regardless of age, preferred traditional medicine over hospital. One factor explaining these preferences may be that the town-born BaYaka are more familiar with Western medicine as they frequently observe farmers using the hospital and selling medicines in the open-air market. Farmers choosing to visit hospitals, instead of consulting the BaYaka for traditional medicine, may weaken some of the prestige associated with using wild plants. We should, nevertheless, note that these are reported preferences and we do not have quantitative data on what percentage of the BaYaka and from which regions use the hospital in town.

One factor that may explain the choice between going to hospital and using traditional medicine is the severity of the illness. For example, some town-born participants said they prefer going to hospital first, but if the treatment is not successful then they use traditional medicine. On the other hand, some forest-born BaYaka indicated that they use traditional medicine primarily, but if the illness is serious they go to hospital. Another factor is the cultural perceptions around specific illnesses. For example, although the town-born BaYaka reported preferring going to hospital over traditional medicine, our field observations suggested that BaYaka might still rely on traditional medicine for the treatment of certain conditions. Both in 2014 and 2018 fieldwork, we observed cases of a specific illness, which the BaYaka referred to as ‘*kole*’. The BaYaka believe that *kole* is caught when a person incautiously steps on an invisible creature that can take the form of different animals and falls from the sky when there is a rainbow. The symptoms are severe as the individuals with the condition lose weight rapidly, have a swollen limb and cannot walk. Our suggestion of taking the sick person to the hospital was refused, as the BaYaka believe that people die in the hospital when they have caught *kole*. Instead, the BaYaka use a plant mixture, which they smear on the sick person and believe that the only treatment for *kole* is to use traditional medicine. If in the past, for severe illnesses like *kole*, people took the sick patient to the hospital as a last resort and the patient died at the hospital, this event might have caused the establishment of the disbelief in hospital treatment.

BaYaka wild plant knowledge is the product of cultural accumulation over hundreds of generations. It may well be the case that some uses of plants have health benefits, and according to a recent report by the World Health Organization (2019), an estimated 80–99% of the population in Congo use indigenous traditional medicine. Our previous findings showed that 77% of the 33 plants used in our interviews had known bioactive properties, some being used by other great apes as medicines, and mothers who used more of the frequently used plants for treating respiratory system disorders had children with higher body mass index (Salali et al., 2016). Therefore, maintenance of wild plant knowledge repertoire is especially important for the BaYaka who do not have easy access to Western medicine. Nevertheless, mortality owing to easily treatable conditions such as diarrhoea and bacterial and respiratory infections are high, especially in infants, and we have come across many individuals suffering from neglected tropical diseases such as yaws and leprosy. For this reason, it is important that these communities have access to Western medicine.

One limitation of our study is that it is not longitudinal, i.e. it does not compare the knowledge of one generation of adults with the next. Our findings, however, indicate that change in the knowledge and use of wild plants has happened within a couple of generations. Pokola used to be a small fishing village surrounded by dense rainforests until the late 1960s, when a logging company was established. The village has since developed into a large market town. This and the fact that we had only one participant older than 45 years who reported being born in town suggest the BaYaka town settlement is relatively new, with no more than two generations having lived there. Therefore, it is likely that loss of knowledge about wild plants has happened within no more than two generations. Nevertheless, our finding that the forest-born BaYaka who were residing in town at the time of study did not differ in their plant knowledge and use from those who were born and residing in forest suggests that cultural loss does not happen within a generation. It is important to note that we did not have information on how long ago those participants who were born in forest moved to town. Therefore, it may be that some forest-born BaYaka were in town temporarily and hence did not differ in their plant use from those who were residing in the forest.

In his reply to Henrich and Norenzeyan's seminal article (2010), Rozin (2010) argues that WEIRD populations may not be as informative when it comes to our understanding of human nature now or in the past, but they are crucial in our understanding of what humans are going to be like in the future. In this paper, we show that the process of global WEIRDing is happening even in the remotest parts of the world. One product of cumulative culture, repertoire of wild plant uses, is being replaced by another product of cumulative culture, namely Western medicine. Similar processes are happening in other indigenous populations. The young Inuit hunters rely more and more on GPS devices rather than orienting themselves by observing wind, snow, animals and sky (Aporta & Higgs, 2005). The Tsimane’ forager–horticulturalists who are living close to towns report fewer plant uses as they perceive the obtaining of other skills to be more valuable for the new socio-economic conditions (Reyes-García, Guèze et al., 2013).

Change in cultural traits in these populations can be seen as adaptive responses to changes in socio-economic and ecological conditions, rather than despairing cultural losses. Although the BaYaka who are born in town may be losing the diversity of their traditional plant knowledge, it may be equally important for their resilience to have the necessary skills to navigate their way in market economies and become an autonomous group who can successfully defend their rights. For the BaYaka who choose to reside in towns permanently, focusing on learning new skills, such as French or mathematics, may be more beneficial in navigating their way through market economies. Flexible cultural responses towards economic, social and political changes for many hunter–gatherer groups may be the one which allows them to be completely autonomous and free in their access to resources, either in the forest or in market towns.

## Data Availability

All data files and associated R codes are available at https://osf.io/udwzr/.

## Supplementary material

For supplementary material accompanying this paper visit http://dx.doi.org/10.1017/ehs.2020.26. click here to view supplementary material
