# Supplementary material for: Global WEIRDing: transitions in wild plant knowledge and treatment preferences in Congo hunter–gatherers
Source: Evol Hum Sci. 2020 Jun 1;2:e24. doi: 10.1017/ehs.2020.26 (PMC10427474; doi:10.1017/ehs.2020.26)
Supplement: Supplementary file 1 [file S2513843X20000262sup001.docx]

# Supplementary information

# Global WEIRDing: Transitions in Wild Plant Knowledge and Treatment Preferences in Congo Hunter-Gatherers

Gul Deniz Salali^1,*^, Mark Dyble^1^, Nikhil Chaudhary^1,2^, Gaurav Sikka^1^, Inez Derkx^1^, Sarai M. Keestra^1,3^, Daniel Smith^1,4^, James Thompson^1^, Lucio Vinicius^1,5^ and Andrea Bamberg Migliano^1,5^

**^1^**Department of Anthropology, University College London, London WC1H 0BW, United Kingdom; ^2^Leverhulme Centre for Human Evolutionary Studies, Department of Archaeology, University of Cambridge, Cambridge, CB2 1QH, United Kingdom; ^3^ Department of Anthropology, Durham University, Durham DH1 3LE, United Kingdom; ^4^Bristol Medical School: Population Health Sciences, University of Bristol, Bristol BS8 2BN, United Kingdom; ^5^Department of Anthropology, University of Zurich, 8057 Zürich, Switzerland

*Correspondence: guldeniz.salali@ucl.ac.uk

## *Measuring plant knowledge and use*

### *Plant list*

At our first campsite, the first author (with her BaYaka translator who translated from French to Mbendjele) asked individuals to list the names of the camp members who are known to have good knowledge of plants. The BaYaka have healers known as *ngangas*. Although families may specialize and are known to be experts in certain treatments, an individual can develop a high reputation and become *nganga* because of his/her skills in healing people. After choosing 15 adults (one of which was the *nganga* of the camp, five females) as informants the first author asked them to list the names of the medicinal plants they use. This initial list consisted of 83 vernacular names. The first authors calculated how many times each of the 83 vernacular names was mentioned by informants. To avoid biasing our questionnaire sample with either plants that are used very frequently or seldom, she chose 33 plant species with mixed use-frequencies. For example, one plant on the list was mentioned by only one informant, whereas another was mentioned by 7 out of 15. After choosing 33 plants to use in our questionnaires, three informants (two from camp one, one from camp three), at different times, walked us around the forest and showed the first author the trees to take photos for identification and to ensure consensus for the vernacular names.

### *Types of uses of plants by the Mbendjele BaYaka hunter-gatherers*

The first author (with her BaYaka translator who translated from French to Mbendjele) asked 219 individuals whether they used each of the 33 plants species on our list. We then categorised the open answers with the help of the Biodiversity Information Standard for economic botany data (Cook, 1995). Although the majority of the uses were for medicinal purposes, we identified some other use categories. For instance, some plant parts were used as poisons to kill monkeys or fish. Some trees were known to have beehives or caterpillars so they were recognized as potential honey or caterpillar reserves. We categorised those answers as foraging related uses. Other uses concerned social norms and beliefs. For example, some plants were used to identify liars: if someone is accused of committing adultery or stealing from someone, the bark of a tree is boiled to make a drink. This drink is believed to be selectively poisonous: they poison liars and leave truthful people well. Other plant uses included making materials such as baskets or rugs, or plants that are consumed as food or are eaten by animals. For full list of plant uses see (Salali et al., 2016).


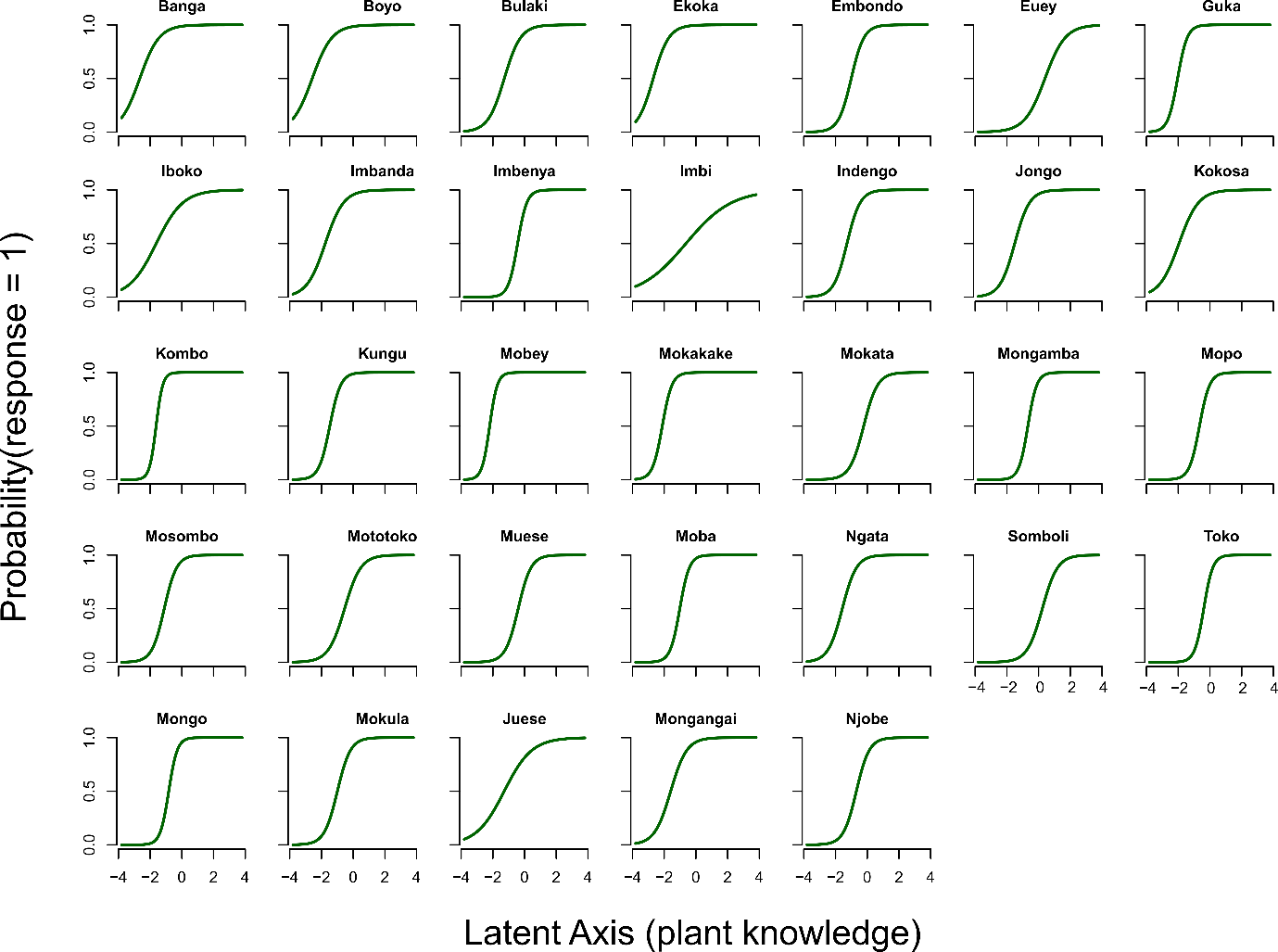
 Supplementary Figure S1: Logistic functions for the probability of knowing about each of the 33 plants as a function of a single latent axis derived from a simple IRT (item response theory) model with no fixed effects. The fact that all relationships are positive is indicative of knowledge of each individual plant mapping well onto a single dimension of overall knowledge.

Supplementary Figure S2: Birth place of the participants by their camp residence.

Supplementary Figure S3: MCA map of each factorial variable based on plant uses. Triangles show the mean of the points of individuals who belonged to a particular category. Pink labels show categories of birth region, orange of age groups, blue of current camp residence and green of sex. The further the category is from the origin, the more different it is compared to the average response pattern.

Supplementary Table S1: ANOVA results of the effects of camp residence (forest vs town), sex and age group on the number of plants that are known (Models 1-1 and 1-2) and used (Models 2-1 and 2-2) by the BaYaka who were born in forest regions. Our analysis of model selection indicated that the models 1-2 and 2-2 were the most parsimonious ones. Children were excluded because there were no children who were born in forest and residing in town.

|  | Forest born forest living vs forest born town living BaYaka | | | | | | | | | | | |
| --- | --- | --- | --- | --- | --- | --- | --- | --- | --- | --- | --- | --- |
|  | **Knowledge score** | | | | | | **Use score** | | | | | |
|  | **Model 1-1** | | | **Model 1-2** | | | **Model 2-1** | | | **Model 2-2** | | |
|  | Df | F value | P value | Df | F value | P value | Df | F value | P value | Df | F value | P value |
| Current camp residence (forest vs town) | 1 | 0.52 | 0.47 |  |  |  | 1 | 1.11 | 0.29 |  |  |  |
| Sex (female, male) | 1 | 10.26 | <0.01 | 1 | 10.34 | <0.01 | 1 | 0.90 | 0.35 | 1 | 0.87194256 | 0.35 |
| Age (young, adult, old) | 2 | 19.12 | <0.001 | 2 | 19.27 | <0.001 | 2 | 31.51 | <0.001 | 2 | 27.2966388 | <0.001 |
| N | 147 | | | | | | | | | | | |

Supplementary Table S2: ANOVA results of the effects of birth region (forest vs town), sex and age group on the number of plants that are known (Models 1) and used (Models 2-1 and 2-2) by the BaYaka who were residing in the town camp at the time of data collection. Our analysis of model selection indicated that the models 1 and 2-2 were the most parsimonious ones. Children and old adults were excluded because there were no children who were born in forest and residing in town; and only one elderly who was born and residing in town at the time of study.

|  | Forest born town living vs town born town living BaYaka | | | | | | | | |
| --- | --- | --- | --- | --- | --- | --- | --- | --- | --- |
|  | **Knowledge score** | | | **Use score** | | | | |  |
|  | **Model 1** | | | **Model 2-1** | | | **Model 2-2** | | |
|  | Df | F value | P value | Df | F value | P value | Df | F value | P value |
| Birth place (forest vs town) | 1 | 21.24 | <0.001 | 1 | 8.06 | <0.01 | 1 | 8.20 | <0.01 |
| Sex (female, male) | 1 | 12.41 | <0.001 | 1 | 0.01 | 0.91 |  |  |  |
| Age (young, adult) | 1 | 7.48 | <0.01 | 1 | 12.21 | <0.01 | 1 | 12.28 | <0.001 |
| N | 52 | | | | | | | | |

**Regression Analyses**

Our multiple regression analyses confirmed our results from ANOVA presented in the main text. Table S3-S4 shows the results from these analyses.

Table S3: Dependent variable: Plant knowledge score. Independent variables: birth place (town as the baseline level), age (old adult as the baseline level), sex (female as the baseline level)

|  | Estimate | Std. Error | t value | P value |
| --- | --- | --- | --- | --- |
| birth_place: ibamba | 4.84 | 1.14 | 4.23 | <0.001 |
| birth_place: minganga | 5.75 | 1.06 | 5.44 | <0.001 |
| birth_place: other | 5.28 | 1.31 | 4.03 | <0.001 |
| Age: adult | -2.81 | 0.83 | -3.39 | <0.001 |
| Age: child | -14.17 | 1.29 | -11.01 | <0.001 |
| Age: young | -6.51 | 1.00 | -6.51 | <0.001 |
| Sex: M | 2.91 | 0.68 | 4.25 | <0.001 |

Table S4: Dependent variable: Plant use score. Independent variables: birth place (town as the baseline level), age (old adult as the baseline level), sex (female as the baseline level)

|  | Estimate | Std. Error | t value | P value |
| --- | --- | --- | --- | --- |
| birth_place: ibamba | 3.51 | 1.45 | 2.43 | <0.05 |
| birth_place: minganga | 5.05 | 1.34 | 3.77 | <0.001 |
| birth_place: other | 3.14 | 1.66 | 1.89 | 0.06 |
| Age: adult | -3.67 | 1.05 | -3.50 | <0.001 |
| Age: child | -17.92 | 1.63 | -11.00 | <0.001 |
| Age: young | -10.48 | 1.27 | -8.28 | <0.001 |
| Sex: M | -0.12 | 0.87 | -0.14 | 0.89 |

**Age Effects on Treatment Method and Preference**

We had individual age estimates, obtained using a Bayesian age estimation method (Diekmann et al., 2017), for the participants in forest camps (n= 50). Using a regression analysis, we analysed whether there is a correlation between age and individual treatment method and preference. Age did not have a significant effect (Table S5 and S6).

Table S5: Regression analysis on age and treatment method (hospital, traditional medicine (tm), pills)

|  | Estimate | Std. Error | t value | P value |
| --- | --- | --- | --- | --- |
| Treatment: pills | -6.05 | 14.40 | -0.42 | 0.68 |
| Treatment: tm | -2.18 | 9.86 | -0.22 | 0.83 |

Table S6: Regression analysis on age and treatment preference (hospital, traditional medicine, hospital first, traditional medicine first)

|  | Estimate | Std. Error | t value | P value |
| --- | --- | --- | --- | --- |
| trad_vs_hospital: hospital_first | 3.09 | 11.66 | 0.26 | 0.79 |
| trad_vs_hospital: tm | 9.74 | 6.12 | 1.59 | 0.12 |
| trad_vs_hospital: tm_first | -0.18 | 7.77 | -0.02 | 0.98 |

**References**

Cook, F. E. M. (1995). *Economic Botany Data Collection Standard. Prepared for the International Working Group on Taxonomic Databases for Plant Sciences (TDWG).* Kew: Royal Botanic Gardens, Kew.

Diekmann, Y., Smith, D., Gerbault, P., Dyble, M., Page, A. E., Chaudhary, N., … Thomas, M. G. (2017). Accurate age estimation in small-scale societies. *Proceedings of the National Academy of Sciences*, *114*(31), 8205–8210.

Salali, G. D., Chaudhary, N., Thompson, J., Grace, O. M., Burgt, X. M. van der, Dyble, M., … Migliano, A. B. (2016). Knowledge-sharing networks in hunter-gatherers and the evolution of cumulative culture. *Current Biology*, *26*(18), 1–6. https://doi.org/10.1016/j.cub.2016.07.015
